# Identification of Tumor Microenvironment-Related Prognostic Genes in Sarcoma

**DOI:** 10.3389/fgene.2021.620705

**Published:** 2021-02-01

**Authors:** Dongjun Dai, Lanyu Xie, Yongjie Shui, Jinfan Li, Qichun Wei

**Affiliations:** ^1^Department of Radiation Oncology, The Second Affiliated Hospital, Zhejiang University School of Medicine, Hangzhou, China; ^2^Department of Clinical Medicine, Fuzhou Medical College of Nanchang University, Jiangxi, China; ^3^Department of Pathology, The Second Affiliated Hospital, Zhejiang University School of Medicine, Hangzhou, China

**Keywords:** sarcoma, tumor microenvironment, TCGA, ESTIMATE algorithms, nomogram, HDAC inhibitors

## Abstract

**Aim:**

Immune cells that infiltrate the tumor microenvironment (TME) are associated with cancer prognosis. The aim of the current study was to identify TME related gene signatures related to the prognosis of sarcoma (SARC) by using the data from The Cancer Genome Atlas (TCGA).

**Methods:**

Immune and stromal scores were calculated by estimation of stromal and immune cells in malignant tumor tissues using expression data algorithms. The least absolute shrinkage and selection operator (lasso) based cox model was then used to select hub survival genes. A risk score model and nomogram were used to predict the overall survival of patients with SARC.

**Results:**

We selected 255 patients with SARC for our analysis. The Kaplan–Meier method found that higher immune (*p* = 0.0018) or stromal scores (*p* = 0.0022) were associated with better prognosis of SARC. The estimated levels of CD4+ (*p* = 0.0012) and CD8+ T cells (*p* = 0.017) via the tumor immune estimation resource were higher in patients with SARC with better overall survival. We identified 393 upregulated genes and 108 downregulated genes (*p* < 0.05, fold change >4) intersecting between the immune and stromal scores based on differentially expressed gene (DEG) analysis. The univariate Cox analysis of each intersecting DEG and subsequent lasso-based Cox model identified 11 hub survival genes (*MYOC*, *NNAT*, *MEDAG*, *TNFSF14*, *MYH11*, *NRXN1*, *P2RY13*, *CXCR3*, *IGLV3-25*, *IGHV1-46*, and *IGLV2-8*). Then, a hub survival gene-based risk score gene signature was constructed; higher risk scores predicted worse SARC prognosis (*p* < 0.0001). A nomogram including the risk scores, immune/stromal scores and clinical factors showed a good prediction value for SARC overall survival (C-index = 0.716). Finally, connectivity mapping analysis identified that the histone deacetylase inhibitors trichostatin A and vorinostat might have the potential to reverse the harmful TME for patients with SARC.

**Conclusion:**

The current study provided new indications for the association between the TME and SARC. Lists of TME related survival genes and potential therapeutic drugs were identified for SARC.

## Introduction

Sarcoma (SARC) is a term used for a heterogeneous group of cancers that originate from somatic mesenchymal tissues. SARC is a rare neoplasm that accounts for less than 1% of newly diagnosed adult cancers (Hui, 2016). The most common therapy for localized SARC to date is surgery in combination with radiation therapy and chemotherapy (Raj et al., 2018). However, a high recurrence rate of nearly 50% has been reported in patients with SARC, and the chemotherapy used for metastatic SARC does not significantly improve the survival of patients. The median overall survival time of metastatic SARC is between 8 and 12 months (Raj et al., 2018). Hence, new therapies are required for the treatment of SARC.

Immunotherapy is an attractive alternative treatment option for SARC. Recently, therapies using immune checkpoint inhibitors, such as anti-cytotoxic T lymphocyte-associated antigen 4 (anti-CTLA-4), and the anti-programmed cell death protein 1 pathway (anti-PD-1/PD-L1) have performed well in the treatment of cancers. However, the efficiency of immune checkpoint inhibitor treatment has been limited in the treatment of SARC (Maki et al., 2013; D’Angelo et al., 2017; Tawbi et al., 2017; Toulmonde et al., 2018).

The immunotherapy response is dependent on complex interactions between the tumor and immune cells within the tumor microenvironment (TME). Various processes within the TME suppress the interactions between tumors and immune effector cells, thus, tumor cells can escape from the attacking immune cells. Therefore, a better understanding of the TME of SARCs is important for improving response to immunotherapy, and will enable the development of more effective therapies (Raj et al., 2018).

The TME comprises various cell types, such as immune, stromal, endothelial, inflammatory, and mesenchymal cells (Hanahan and Coussens, 2012). Among these, immune and stromal cells are two major non-cancer cell types found in the TME that are associated with the prognosis of cancers (Garcia-Gomez et al., 2018). Recently, with the development of sequencing technology and the establishment of large molecular databases such as The Cancer Genome Atlas (TCGA), many algorithms were developed to exploit the TME (Carter et al., 2012; Yoshihara et al., 2013). For example, the estimation of stromal and immune cells in malignant tumor tissues using expression data (ESTIMATE) algorithm uses gene expression signatures to infer the infiltration level of stromal and immune cells in tumor samples by calculating stromal and immune scores (Yoshihara et al., 2013). The ESTIMATE algorithm has been applied to several cancer types and it has been identified that high immune or stromal scores were associated with favorable prognoses in osteosarcoma (Hong et al., 2020) and cervical squamous cell carcinoma (Pan et al., 2019), and unfavorable prognosis in gastric cancer (Wang et al., 2019), bladder cancer (Zhang et al., 2020), and acute myeloid leukemia (Ni et al., 2019). However, the ESTIMATE algorithms have not been previously used to explore the association between immune and stomal cells and the prognosis of SARC in adults.

This study aims to apply the ESTIMATE algorithm to the SARC RNA sequencing (RNA-seq) data from TCGA database. This will enable the construction of a TME related gene signature to predict the overall survival of patients with SARC.

## Materials and Methods

### Database and ESTIMATE Algorithm Application

The RNA-seq read counts and clinical data of patients with SARC were taken from TCGA project. We downloaded the relative data from the Xena database (Goldman et al., 2019). The patients with both read count data and survival information were included in the following analyses. Raw counts data were normalized by the TMM method from the “edgeR” R package and then transformed with the voom method from the “limma” R package. The ESTIMATE algorithm was applied to the selected patients with SARC to calculate the immune and stromal scores via the “estimate” R package.

The Kaplan–Meier (KM) method was used to draw the survival curve. A log-rank test was applied to the KM plot. The best cutoff value was calculated to grade the SARC groups based on the level of immune or stromal scores. The stratified high and low groups were then used for the following analyses.

### Tumor Immune Estimation Resource Analysis

To explore the association between immune scores or stromal scores and the immune cells, the SARC fragments per kilobase of transcript per million mapped reads data from the Xena database were transformed into transcripts per million and subjected to tumor immune estimation resource (TIMER) analysis (Li et al., 2020). Six tumor-infiltrating cell populations were analyzed: B cells, CD4+ T cells, CD8+ T cells, neutrophils, macrophages, and dendritic cells. KM analysis, with the establishment of optimal cutoff value, was performed to access the association between immune cell type and the prognosis of patients with SARC.

### Identification of Differentially Expressed Genes Between High and Low Immune or Stromal Score Groups

The “limma” R package was used to find the differentially expressed genes (DEGs) between high and low immune or stromal score groups. The DEGs were defined as the genes with a fold change > 4 and adjusted *p*-value < 0.05 based on the results of the “limma” analysis. The results of the DEG analysis were visualized using volcano plots and heatmaps. The intersecting parts of the DEGs analyses of the immune and stromal scores groups were calculated and grouped by Venn diagram. The intersecting genes would then be used for functional analysis.

### Functional Analysis of the Intersecting DEGs of Immune or Stromal Score Groups

Functional analyses were performed for intersecting DEGs of immune score or stromal score groups. Kyoto Encyclopedia of Genes and Genomes (KEGG) analysis and gene ontology (GO) analysis, which consists of biological processes (BP), cellular components (CC), and molecular functions (MF), were used. The *p*-value < 0.05 and *q*-value < 0.05 were set as the cutoff value.

### Survival Analysis of Intersecting DEGs and Clinical Predictive Model Construction

Univariate Cox analysis was applied for the high or low expression groups (Stratified by median value) of each DEG. DEGs with a *p*-value < 0.05 were considered to be a survival related DEG. The least absolute shrinkage and selection operator (lasso) analysis was performed to select the hub survival related genes. The selected survival related genes from lasso analysis were then used to calculate the risk score, which was calculated as (*βi* × Exp*i*) (*i* = the number of hub survival related genes). The optimal cutoff value of the risk score was calculated, following which a KM plot was drawn. The area under the receiver operating characteristic curve (AUC) was calculated for the 1-year, 3-year, and 5-year survival prediction of patients with SARC.

A multivariate Cox model-based nomogram was constructed for the 1-year, 3-year, and 5-year predictions of the overall survival of patients with SARC. The internal validation was determined by discrimination and calibration with 1,000 bootstraps. The C-index was calculated and the calibration curve was plotted.

### Drug Identification Analysis

Connectivity Map (CMap) analysis uses a reference database containing drug-specific gene expression profiles and compares it with a disease-specific gene signatures. This enables accurate drug identification for certain disease phenotypes (Lamb, 2007; Musa et al., 2018). The CMap dataset consists of cellular signatures that catalog transcriptional responses of human cells to chemical and genetic perturbation, which are then widely used as reference profiles for connectivity mapping analysis (Subramanian et al., 2017). In this study, we used the R package “Dr. Insight” to perform CMap analysis. It provides a connectivity mapping method to connect drugs (compounds) in the CMap dataset with query data (disease phenotype, such as immune and stromal scores). The results of the *t*-test statistic scores from the “limma” analysis were used as input data for this evaluation. We identified the drugs that targeted patients with SARC with lower immune or stromal scores that had worse survival. The drugs with a false discovery rate (FDR) < 0.1 were considered as key targets for the therapy of patients with SARC with lower immune or stromal scores.

### Statistical Analysis

All the statistical analyses were performed using R-4.0.2. The “survminer” package was used for the KM analysis. The “immunedeconv” package was used for TIMER analysis. The “VennDiagram” package was used to draw the Venn diagram. The “pheatmap” package was used to plot the heatmap. The “clusterProfiler” package was used for the functional analyses. The “glmnet” package was used to perform the lasso analysis. The “ROCR” package was used for the AUC analysis. The “rms” package was used for the nomogram construction and validation. The “ggplot2” package was used to draw KM plots, box plots, volcano plots and histogram. For comparisons between two groups, Wilcoxon analysis was performed, while for comparisons among three or more groups, Kruskal–Wallis analysis was applied.

## Results

### Data Selection and ESTIMATE Algorithm Results

**FIGURE 1:**
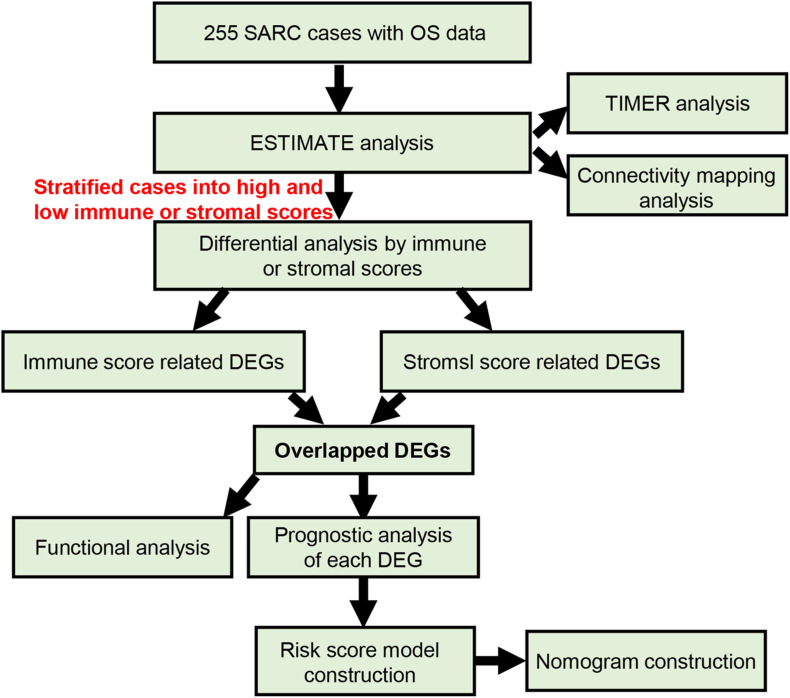
The flow chart of current study.

As shown in Figure 1, we selected 255 patients with SARC with read counts data and clinical information. The detailed clinical information of the included patients is shown in Table 1. There were 139 (54.51%) patients 60 years or older and 116 (45.49%) patients under 60 years old, 139 (54.51%) were female and 116 (45.49%) were male, and 18 (7.06%) were African American patients and 223 were (87.45%) Caucasian American patients. The primary disease diagnosis for the patients were 56 dedifferentiated liposarcomas (21.96%), 25 fibromyxosarcomas (9.80%), 100 leiomyosarcomas (39.22%), 12 malignant fibrous histiocytomas (4.71%), and 33 undifferentiated SARCs (12.94%). The SARC disease type were 40 fibromatous neoplasms (15.69%), 58 lipomatous neoplasms (22.75%), 103 myomatous neoplasms (40.39%), 35 soft tissue tumors and SARC (13.73%), and 10 synovial-like neoplasms (3.92%). The primary site distribution was in connective, subcutaneous and other soft tissues in 114 (44.71%) patients, the retroperitoneum and peritoneum in 98 (38.43%) patients, and the uterus in 27 (10.59%) patients.

**TABLE 1:** The clinical characteristics of patients with SARC.

Characteristics	No. of patients	%
**Age**		
High	139	0.545
Low	116	0.455
**Gender**		
Female	139	0.545
Male	116	0.455
**Race**		
African American	18	0.071
Caucasian American	223	0.875
Other	14	0.055
**Primary diagnosis**		
Dedifferentiated liposarcoma	56	0.22
Fibromyxosarcoma	25	0.098
Leiomyosarcoma, NOS	100	0.392
Malignant fibrous histiocytoma	12	0.047
Undifferentiated sarcoma	33	0.129
Other	29	0.114
**Disease type**		
Fibromatous Neoplasms	40	0.157
Lipomatous Neoplasms	58	0.227
Myomatous Neoplasms	103	0.404
Soft Tissue Tumors and Sarcomas, NOS	35	0.137
Synovial-like Neoplasms	10	0.039
Nerve Sheath Tumors	9	0.035
**Primary site**		
Connective, subcutaneous and other soft tissues	114	0.447
Retroperitoneum and peritoneum	98	0.384
Uterus, NOS	27	0.106
Other	16	0.063
**Stromal Score**		
High	159	0.624
Low	96	0.376
**Immune Score**		
High	166	0.651
Low	89	0.349
**Risk_Scores**		
High	74	0.29
Low	181	0.71

**FIGURE 2:**
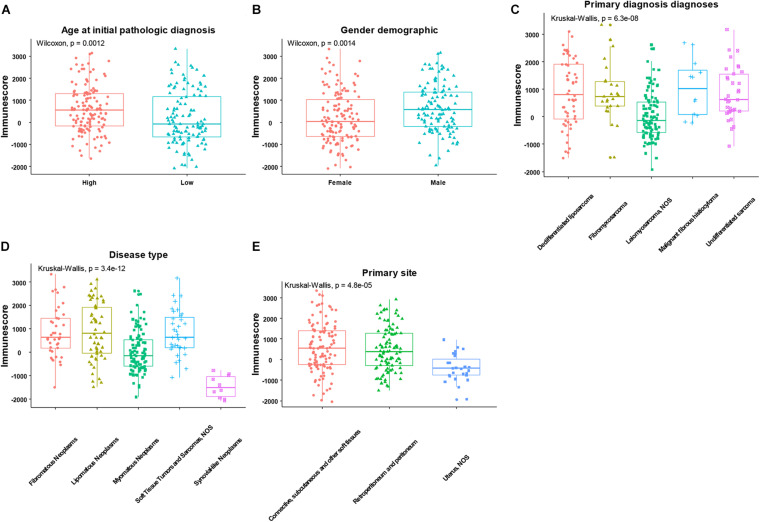
The significant associations between immune score and the clinical factors of patients with SARC. The age **(A)**, gender **(B)**, primary diagnosis **(C)**, disease type **(D)**, and primary site **(E)** were significantly associated with the immune scores calculated by ESTIMATE algorithm.

After using the ESTIMATE algorithm, the immune scores ranged from −2088.757 to 3342.350, while the stromal scores ranged from −1238.948 to 2525.174. The detailed immune scores and stromal scores are listed in Supplementary Table 1. We found that both the immune and stromal scores were significantly associated with age, gender, primary diagnosis, disease type, and primary site (Figure 2 and Supplementary Figure 1, *p* < 0.05). In detail, the immune and the stromal scores were lower in younger patients with SARC than in old patients (Figure 2A and Supplementary Figure 1A), lower in females than males (Figure 2B and Supplementary Figure 1B), lower in leiomyosarcoma and undifferentiated SARCs than other primary diagnosed SARCs (Figure 2C and Supplementary Figure 1C), lower in myomatous neoplasms and synovial-like neoplasms than other disease types of SARC (Figure 2D and Supplementary Figure 1D), and lower in the uterus than other primary sites of SARC (Figure 2E and Supplementary Figure 1E). Besides this, we observed that there were no associations between immune scores or stromal scores and the SARC characteristics that comprised race, tumor total necrosis percent, tumor depth, person neoplasm cancer status, mitotic count, metastatic diagnosis, local disease recurrence, and leiomyosarcoma histologic subtype and margin status (Supplementary Figures 2, 3).

**FIGURE 3:**
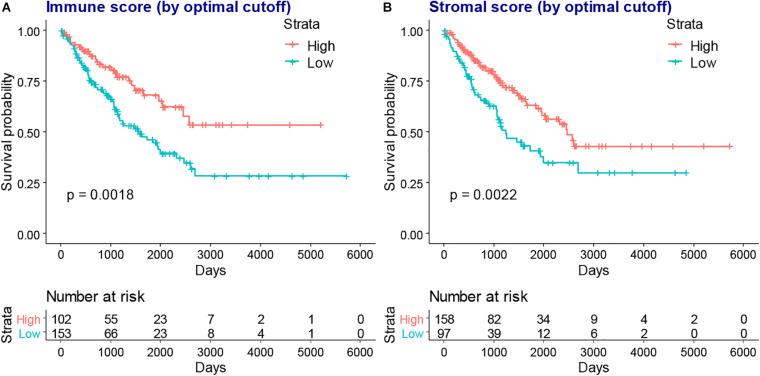
The KM plot of immune scores or stromal scores and the overall survival of patients with SARC. The lower immune scores **(A)** and stromal scores **(B)** indicated worse overall survival in the KM plot model which stratified by optimal cutoff value.

We next evaluated the association between immune and stromal scores and the prognosis of patients with SARC, the optimal cutoff values were evaluated for KM analysis. We found that lower immune or stromal scores were associated with poorer overall survival of patients with SARC (Figure 3).

### Association Between Infiltration Level of Immune Cells and the SARCs Immune or Stromal Scores

**FIGURE 4:**
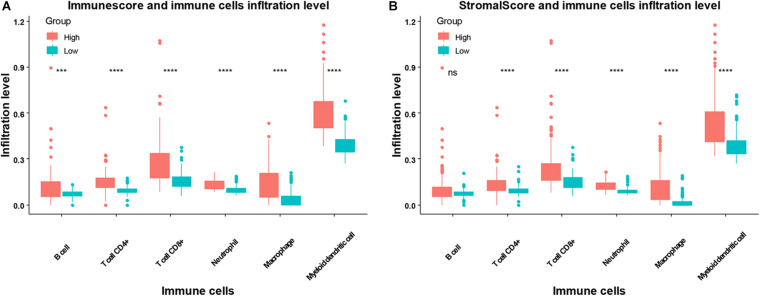
The association between infiltration level of immune cells and the immune scores/stromal scores in patients with SARC. The infiltration levels of B cells, CD4+ T cells, CD8+ T cells, neutrophils, macrophages, and myeloid dendritic cell in patients with SARC according to **(A)** immune scores and **(B)** stromal scores.

**FIGURE 5:**
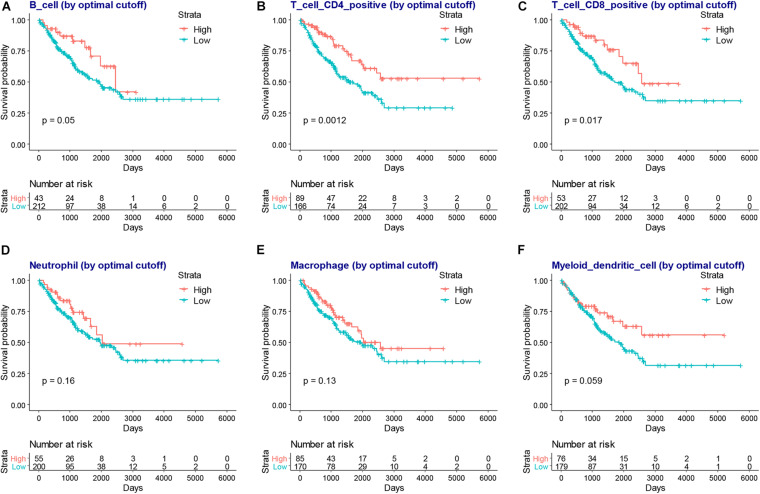
The association between infiltration level of immune cells and the overall survival of patients with SARC. The KM plot of infiltration levels of B cells **(A)**, CD4+ T cells **(B)**, CD8+ T cells **(C)**, neutrophils **(D)**, macrophages **(E)**, and myeloid dendritic cells **(F)** and the overall survival of patients with SARC.

We next compared the infiltration level of immune cells between the higher or lower immune or stromal scores groups. We found CD4+ T cells, CD8+ T cells, neutrophils, macrophages, and dendritic cells were higher in the high immune or stromal score groups, while B cell infiltration was only higher in the immune score group (Figure 4). Moreover, we explored the association between the infiltration level of immune cells and the overall survival of patients with SARC (Figure 5). We found that CD4+ and CD8+ T cells were higher in patients with better overall survival (Figures 5B,C).

### Comparison of Gene Expression Profile by Immune and Stromal Scores

**FIGURE 6:**
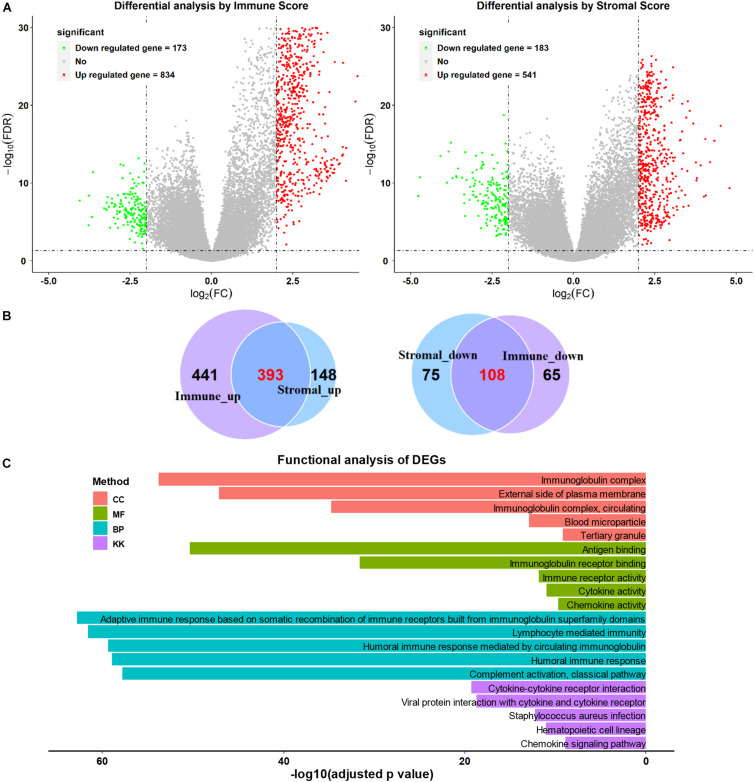
Comparison of gene expression profiles with higher or lower immune scores or stromal scores in SARC. **(A)** The volcano plots were used to describe the differential expressed genes of immune scores and stromal scores. **(B)** The Venn plots were performed to reveal the intersections of up-regulated genes and down-regulated genes. **(C)** The top GO analyses terms and KEGG pathways terms enriched by the intersect DEGs.

We next separated the patients with SARC into two groups according to their immune score level or stromal score level. The high and low immune or stromal score groups were then used for DEG analysis. Heatmaps showed distinct gene expression profiles of high and low immune or stromal score groups (Supplementary Figure 4). The volcano plots showed that there were 834 upregulated genes and 173 downregulated genes in the immune score groups (Figure 6A and Supplementary Table 2), and there were 541 upregulated genes and 183 downregulated genes in stromal score groups (Figure 6A and Supplementary Table 3). The Venn diagram shows that there were 393 upregulated genes and 108 downregulated genes in the intersecting part between the groups (Figure 6B). These DEGs common to both groups were then used for the functional analysis.

### Functional Analysis of Intersecting DEGs

As shown in Figure 6C, functional enrichment GO and KEGG analyses were applied for the intersected DEGs. The top five GO (biological process, cellular component, and molecular function) and KEGG terms were included. The detailed GO and KEGG analysis results are listed in Supplementary Tables 4–7.

The GO analysis showed the intersected DEGs were related to immunoglobulin related terms; such as immunoglobulin complex, immunoglobulin receptor binding, and immunoglobulin immune responses. In addition, the intersected DEGs were associated with KEGG terms like cytokine-cytokine receptor interaction, viral protein interaction with cytokine-cytokine receptor (Figure 6C). Furthermore, the B cell and T cell activation related terms were also enriched (Supplementary Tables 4–7).

### Survival Analysis of Intersecting DEGs and Clinical Predictive Model Construction

To reveal the relationship of intersecting DEGs and the prognosis of SARC, we performed a univariate Cox analysis for each gene. There were 140 SARC survival related DEGs identified (Supplementary Table 8).

Among them, there were 138 survival favorable genes, of which 135 were upregulated in the higher immune or stromal score groups, and there were 2 survival unfavorable genes, which were both downregulated in the higher immune or stromal score groups. This was consistent with the results that patients with SARC with higher immune or stromal scores had better overall survival.

**FIGURE 7:**
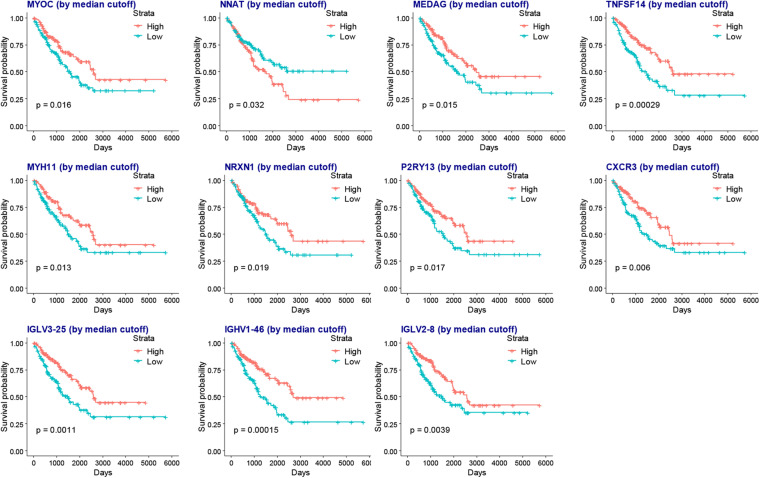
The KM plots of lasso selected survival genes.

**FIGURE 8:**
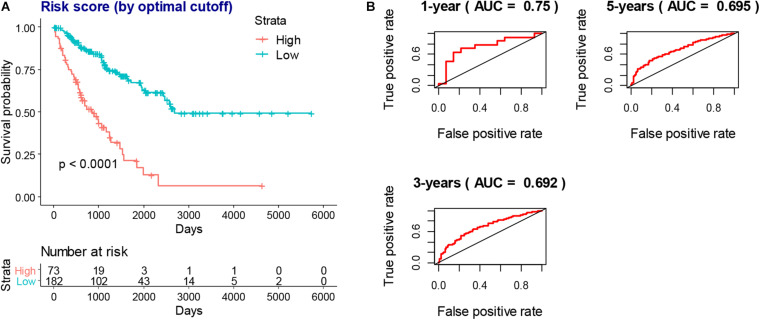
The prognostic value of risk score model. **(A)** The higher risk scores indicated worse overall survival of SARC patients in KM models which stratified by best cutoff value. **(B)** The AUC plots of risk scores to predict the 1-year, 3-year, and 5-year overall survival of SARC patients.

**FIGURE 9:**
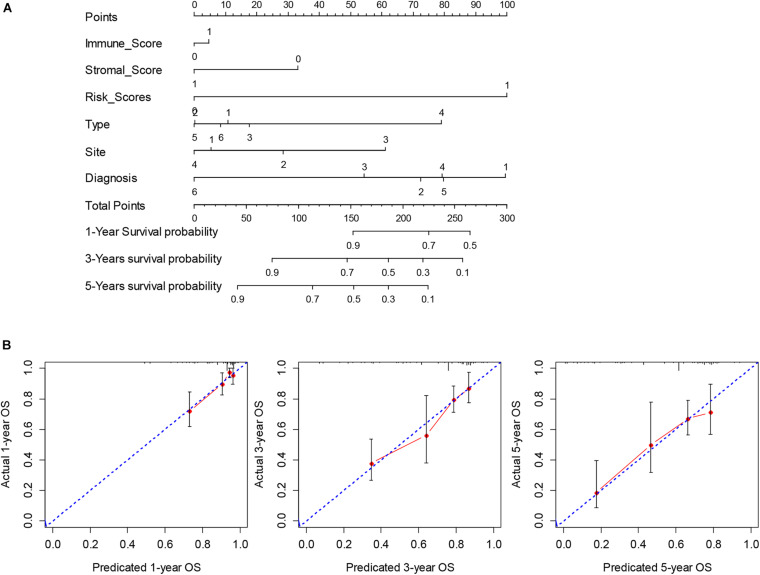
The construction and validation of nomogram. **(A)** The multivariate Cox model based nomogram of SARC patients; Immune_Score: 0 = Low and 1 = High; Stromal_Score: 0 = Low and 1 = High; Risk_Score: 0 = Low and 1 = High; Type: 1 = Fibromatous Neoplasms, 2 = Lipomatous Neoplasms, 3 = Myomatous Neoplasms, 4 = Nerve Sheath Tumors, 5 = Soft Tissue Tumors and Sarcomas (Not Otherwise Specified) and 6 = Synovial-like Neoplasms; Diagnosis: 1 = Dedifferentiated liposarcoma, 2 = Fibromyxosarcoma, 3 = Leiomyosarcoma (Not Otherwise Specified), 4 = Malignant fibrous histiocytoma, 5 = Undifferentiated sarcoma and 6 = Others; Site: 1 = Connective, subcutaneous and other soft tissues, 2 = Retroperitoneum and peritoneum, 3 = Uterus (Not Otherwise Specified) and 4 = Others; **(B)** the calibration plots for the internal validation of current nomogram, the *x*-axis represents the nomogram predicted overall survival and the *y*-axis represents the actual overall survival of patients with SARC.

To obtain a more interpretable prognostic model, we performed a variable selection process by using the lasso-based Cox model. The genes of *MYOC*, *NNAT*, *MEDAG*, *TNFSF14*, *MYH11*, *NRXN1*, *P2RY13*, *CXCR3*, *IGLV3-25*, *IGHV1-46*, and *IGLV2-8* were selected as they had a non-zero value of coefficients (Supplementary Table 9). Among these genes, the increased expression of *NNAT* was associated with worse overall survival, and the elevated expression of *MYOC*, *MEDAG*, *TNFSF14*, *MYH11*, *NRXN1*, *P2RY13*, *CXCR3*, *IGLV3-25*, *IGHV1-46*, and *IGLV2-8* were associated with better overall survival (Figure 7). These lasso selected survival genes were then used to construct a risk score model. We found that the higher risk scores were strongly associated with worse overall survival of patients with SARC (Figure 8A). The AUC plots showed the risk score model was predictable (1-year, AUC = 0.75; 3-year, AUC = 0.692; 5-year, AUC = 0.695; Figure 8B). Furthermore, we built a Cox based nomogram for patients with SARC (Figure 9A). The calibration plots showed the risk model predicted the overall survival of patients with SARC well (Figure 9B). The C-index for the nomogram was 0.716.

### Exploration of Potential Drug Targets

Utilizing CMap analysis, we identified the drugs that had potential therapeutic effects on patients with SARC that had low survival rates. The results showed that the histone deacetylases (HDAC) inhibitors trichostatin A and vorinostat were key drugs that might have therapeutic value (Table 2, *p*-value < 0.05, FDR < 0.1).

**TABLE 2:** Key drugs that had potential therapeutic effects on SARC patients with lower immune or stromal scores.

Drug	*p* value	FDR
**Immune score based**		
trichostatin A_MCF7	2.67E-38	9.57E-35
trichostatin A_PC3	8.45E-28	1.51E-24
vorinostat_MCF7	1.38E-05	0.016
**Stromal score based**		
trichostatin A_MCF7	8.49E-39	3.04E-35
trichostatin A_PC3	2.80E-31	5.03E-28
vorinostat_MCF7	4.93E-05	0.059

## Discussion

Using the ESTIMATE algorithm, we found that patients with SARC with higher immune or stromal scores had better overall survival. We also identified that higher immune or stromal scores represented higher infiltrating levels of CD4+ T cells, CD8+ T cells, neutrophils, macrophages, and dendritic cells. Furthermore, we observed that CD4+ T cells and CD8+ T cells were strongly associated with the survival of patients with SARC.

CD4+ and CD8+ T cell responses play a central role in the elimination of cancer cells (Ostroumov et al., 2018). A previous study found that a higher level of infiltration of CD8+ lymphocytes into synovial SARC was associated with a favorable overall patient survival (Oike et al., 2018). Moreover, patients with cutaneous angiosarcoma with higher levels of CD8+ lymphocytes in primary tumors survived longer when compared with patients with less of these cells. Furthermore, the CD8+ lymphocytes also correlated with a distinct metastasis-free period (Fujii et al., 2014). Another study revealed that the immune checkpoint therapy-mediated rejection of a nonimmunogenic SARC requires both CD4+ and CD8+ T cells (Alspach et al., 2019). Our findings confirmed the importance of CD4+ and CD8+ T cells in the progression of SARC.

The immune or stromal high and low groups’ scores revealed 501 TME related DEGs. According to the enrichment analyses, these DEGs were associated with immunoglobulin related GO terms. Previously, the administration of immunoglobulin G has been shown to be able to inhibit cancer growth (Xu et al., 2019), furthermore, this specific immunoglobulin isotype (IgG) was also found to be associated with cancer prognosis (Hsu et al., 2019; Isaeva et al., 2019). In the lasso-based Cox model, the increased expression of immunoglobulin related genes *IGLV3-25*, *IGHV1-46*, and *IGLV2-8* were also identified to be hub genes associated with better survival, providing new insights into the relationship between immunoglobulins and SARC prognosis. Moreover, we also found that the high levels of expression of *NNAT* and the low levels of expression of *MYOC*, *MEDAG*, *TNFSF14*, *MYH11*, *NRXN1*, *P2RY13*, and *CXCR3* were associated with favorable overall survival of patients with SARC. Most of these genes were previously identified to be related to cancer prognosis, which is consistent with our findings. NNAT is a proteolipid involved in cation homeostasis. Its high expression was found to be associated with poor outcomes in a series of different cancers (Nass et al., 2017) MYOC is a skeletal muscle hypertrophy-promoting protein that was found to be downregulated in multiple cancer cachexia mouse models. The loss of MYOC in these models could induce phenotypes such as muscle fiber atrophy, sarcolemmal fragility, and impaired muscle regeneration (Judge et al., 2020). *MEDAG* is a gene involved in processes that promote adipocyte differentiation. Previously it was characterized to have a lower abundance in ovarian cancer ascites extracellular vesicles (Yamamoto et al., 2018) and ovarian cancer cells (Yeung et al., 2013). TNFSF14 is a protein primarily expressed in activated T cells, activated natural killer cells, and immature dendritic cells. It functions by stimulating effector cell functions and encouraging antitumor CD8+ T cells to enter tumors, aiding in the establishment of anti-tumoral memory (Skeate et al., 2020). *MYH11* encodes a protein that participates in muscle contraction through the hydrolysis of adenosine triphosphate; its expression levels are downregulated in several types of cancers (Alhopuro et al., 2008; Nie et al., 2020). *NRXN1* encodes a transmembrane protein that functions as a cell adhesion molecule in synaptic transmission (Reissner et al., 2008). Its high expression was observed to be associated with favorable overall survival of patients with oral cancers (Hirohata et al., 2018). *P2RY13* encodes a protein belongs to the family of G-protein coupled receptors, moreover, its high expression demonstrated significantly higher overall survival rates in patients with breast cancer (Xu et al., 2020) and lung adenocarcinoma (Fan et al., 2020).

After the survival analysis of TME related DEGs and the lasso based variable selection analysis, a risk score model was constructed with the key survival genes. The risk score model showed good predictions for the overall survival of patients with SARC. Furthermore, we combined the risk model with immune and stromal scores and the clinical variables to build a nomogram for the prognosis of patients with SARC. The nomogram was validated by discrimination and calibration procedures; it is the first nomogram built for the patients with SARC that includes immune related gene signatures.

We found that trichostatin A and vorinostat, which are pan-HDAC inhibitors of class I and II HDACs, had a potential therapeutic effect on the patients with lower immune or stromal scores and worse survival. HDACs play a critical role in the regulation of transcription by promoting the deacetylation of histone proteins (Kulka et al., 2020). The upregulation of specific HDACs have been found in different cancers, including SARC (Tang et al., 2017; Banik et al., 2019). HDAC inhibitors have been shown to have anti-inflammatory properties that can impact cancer therapy (Hull et al., 2016). HDAC inhibitors are closely associated with immunotherapy; they can enhance the expression of cancer antigens, decrease immunosuppressive cell populations like the myeloid-derived suppressor cell, regulate specific suppressive pathways, and induce specific chemokine expression on T cells (Banik et al., 2019). In SARCs, the HDAC inhibitors showed multiple tumor inhibitory effects that included upregulating tumor suppressor genes, downregulating oncogenes, promoting apoptosis (Su et al., 2010) and cell cycle arrest (Sakimura et al., 2005), decreasing invasion, metastasis and angiogenesis (Ailenberg and Silverman, 2003; Lee et al., 2015), inducing reactive oxygen species (ROS) production (Laporte et al., 2017), autophagy (Yamamoto et al., 2008), and cell differentiation (Di Pompo et al., 2015). Our study found that trichostatin A and vorinostat had the potential to reverse the lower immune status of the TME in SARCs and thereby improve the survival of patients; providing new insights into the relationship between HDAC inhibitors and SARC.

Our study had certain limitations. First, due to the lack of RNA seq or microarray SARC data, we only included TCGA data. However, we expect our results to be externally validated with future SARC genomic data. Secondly, the underlying mechanisms of SARC and the key survival genes and HDAC inhibitors should be further explored and characterized.

## Conclusion

In summary, our study found that high immune and stromal cell infiltration levels were associated with better SARC prognosis. The risk gene signature related nomogram is a useful predictive tool for the overall survival of patients with SARC. Finally, we found that two HDAC inhibitors, trichostatin A and vorinostat, may have potential therapeutic value for patients with SARC and suggest this relationship be further investigated.

## Data Availability Statement

The original contributions presented in the study are included in the article/Supplementary Material, further inquiries can be directed to the corresponding author/s.

## Author Contributions

QW and DD designed the study. DD and LX analyzed the data and wrote the manuscript which was checked by QW. YS and JL collected the data. All authors contributed to the article and approved the submitted version.

## Conflict of Interest

The authors declare that the research was conducted in the absence of any commercial or financial relationships that could be construed as a potential conflict of interest.

## Supplementary Material

Supplementary Figure 1 The significant associations between stromal score and the clinical factors of patients with SARC. The age **(A)**, gender **(B)**, primary diagnosis **(C)**, disease type **(D)**, and primary site **(E)** were significantly associated with the stromal scores calculated by ESTIMATE algorithm.

Supplementary Figure 2 The clinical factors of patients with SARC that were not associated with the immune scores. There were no association between the immune scores and the clinical factors of SARC, which comprised race, tumor total necrosis percent, tumor depth, person neoplasm cancer status, mitotic count, metastatic diagnosis, local disease recurrence, leiomyosarcoma histologic subtype, and margin status.

Supplementary Figure 3 The clinical factors of SARC that were not associated with the stromal scores. There were no association between the stromal scores and the clinical factors of SARC, which comprised race, tumor total necrosis percent, tumor depth, person neoplasm cancer status, mitotic count, metastatic diagnosis, local disease recurrence, leiomyosarcoma histologic subtype, and margin status.

Supplementary Figure 4 The heatmap of DEGs profiles between groups of high and low immune or stromal scores.

Supplementary Table 1 The immune scores and stromal scores of 255 patients with SARC.

Supplementary Table 2 The DEGs between high and low immune score groups.

Supplementary Table 3 The DEGs between high and low stromal score groups.

Supplementary Table 4 The functional analysis of overlapped DEGs with GO BP items.

Supplementary Table 5 The functional analysis of overlapped DEGs with GO CC items.

Supplementary Table 6 The functional analysis of overlapped DEGs with GO MF items.

Supplementary Table 7 The functional analysis of overlapped DEGs with KEGG items.

Supplementary Table 8 The significant survival related DEGs by univariate Cox analysis.

Supplementary Table 9 The coefficients of each gene after the lasso analysis.
