# Supplementary figures and images for: Identification of Tumor Microenvironment-Related Prognostic Genes in Sarcoma
Source: Front Genet. 2021 Feb 1;12:620705. doi: 10.3389/fgene.2021.620705 (PMC7882740; doi:10.3389/fgene.2021.620705)

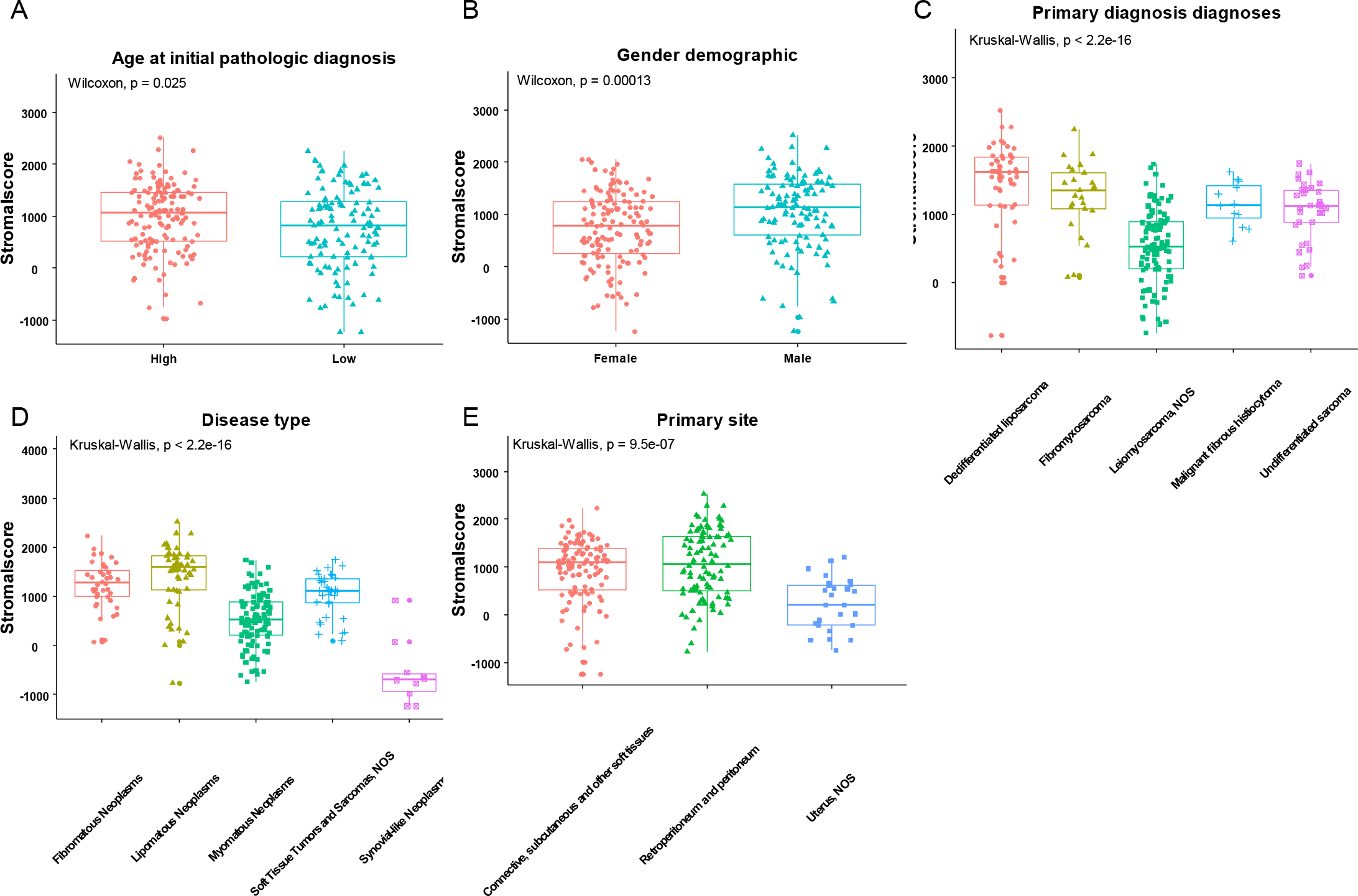

Supplement: Supplementary Figure 1 — The significant associations between stromal score and the clinical factors of patients with SARC. The age (A), gender (B), primary diagnosis (C), disease type (D), and primary site (E) were significantly associated with the stromal scores calculated by ESTIMATE algorithm. [file Image_1.TIF]

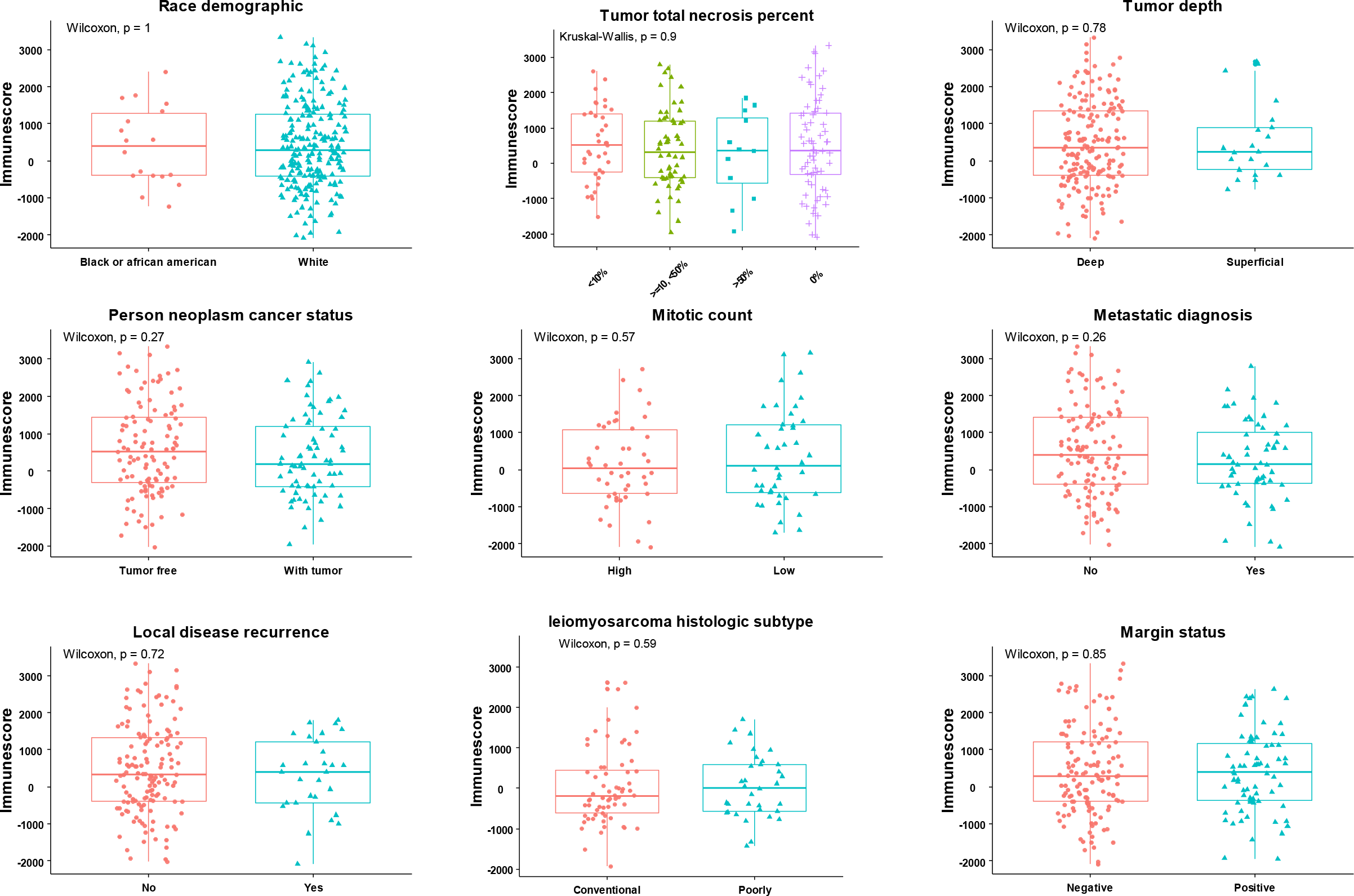

Supplement: Supplementary Figure 2 — The clinical factors of patients with SARC that were not associated with the immune scores. There were no association between the immune scores and the clinical factors of SARC, which comprised race, tumor total necrosis percent, tumor depth, person neoplasm cancer status, mitotic count, metastatic diagnosis, local disease recurrence, leiomyosarcoma histologic subtype, and margin status. [file Image_2.TIF]

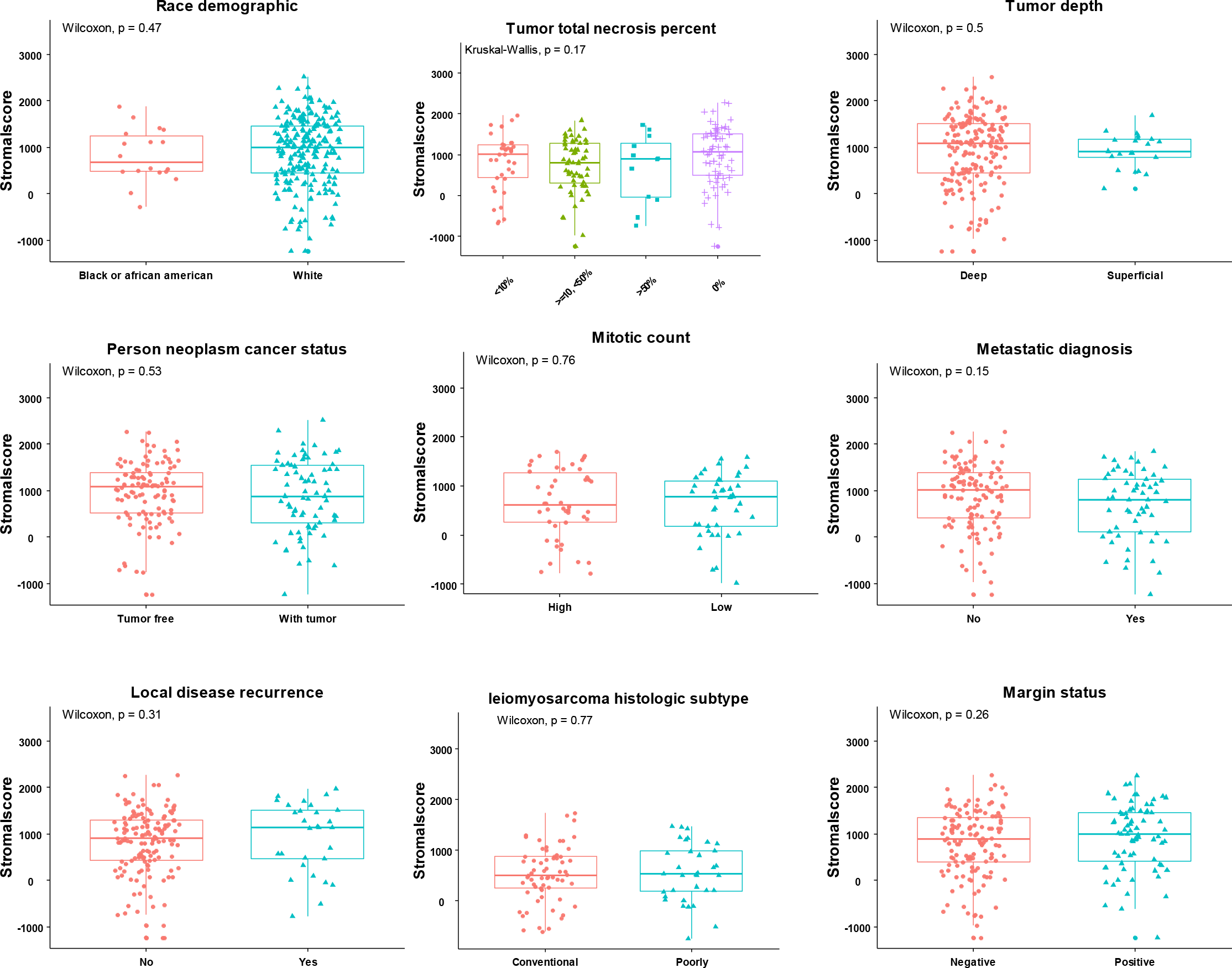

Supplement: Supplementary Figure 3 — The clinical factors of SARC that were not associated with the stromal scores. There were no association between the stromal scores and the clinical factors of SARC, which comprised race, tumor total necrosis percent, tumor depth, person neoplasm cancer status, mitotic count, metastatic diagnosis, local disease recurrence, leiomyosarcoma histologic subtype, and margin status. [file Image_3.TIF]

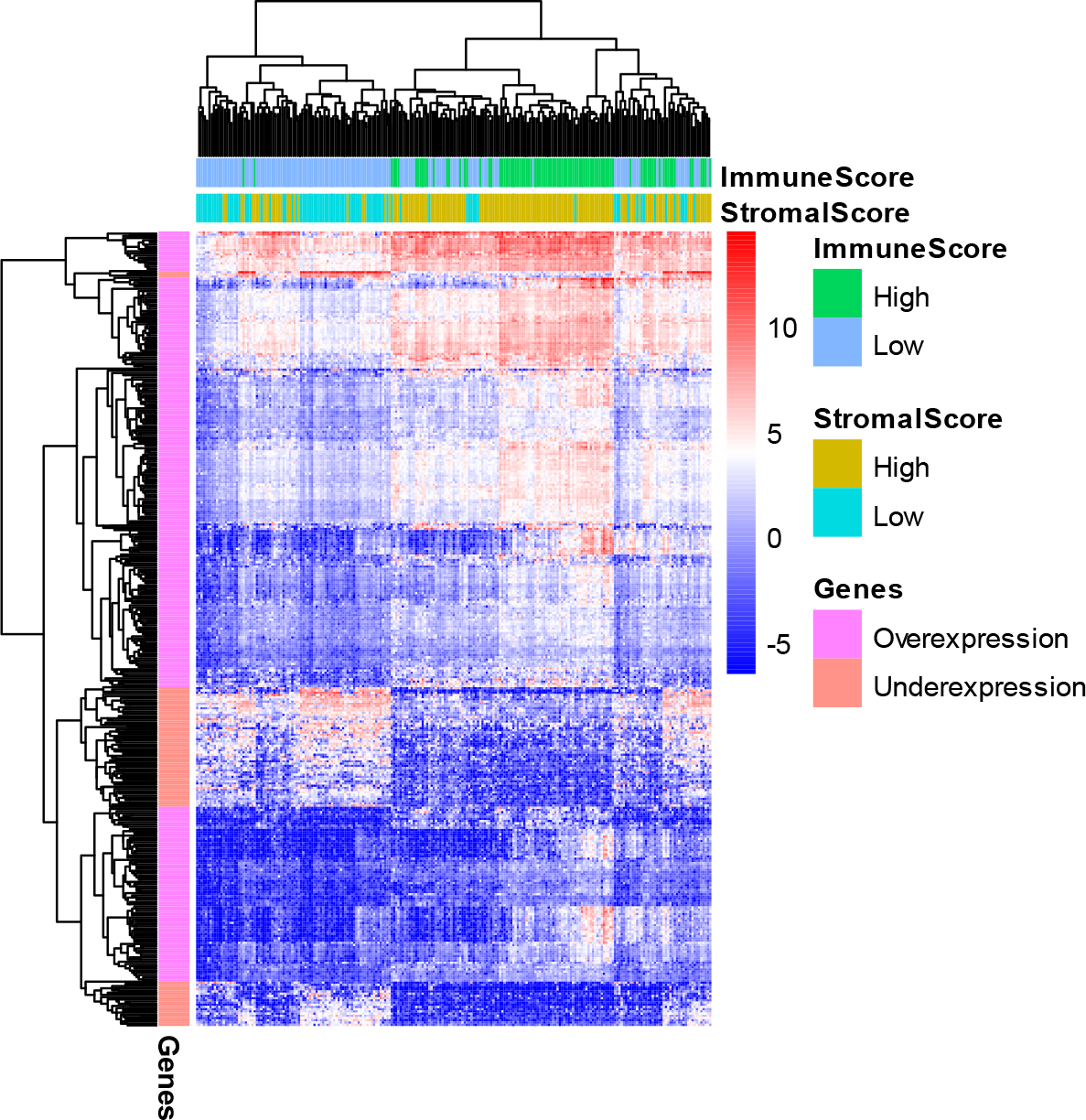

Supplement: Supplementary Figure 4 — The heatmap of DEGs profiles between groups of high and low immune or stromal scores. [file Image_4.TIF]
